# Disorder-specific brain volumetric abnormalities in Attention-Deficit/Hyperactivity Disorder relative to Autism Spectrum Disorder

**DOI:** 10.1371/journal.pone.0241856

**Published:** 2020-11-09

**Authors:** Ariadna Albajara Sáenz, Peter Van Schuerbeek, Simon Baijot, Mathilde Septier, Nicolas Deconinck, Pierre Defresne, Véronique Delvenne, Gianfranco Passeri, Hubert Raeymaekers, Hichem Slama, Laurent Victoor, Eric Willaye, Philippe Peigneux, Thomas Villemonteix, Isabelle Massat

**Affiliations:** 1 Neuropsychology and Functional Neuroimaging Research Group (UR2NF) at the Centre for Research in Cognition and Neurosciences (CRCN), Université Libre de Bruxelles (ULB), Brussels, Belgium; 2 Department of Radiology, Universitair Ziekenhuis Brussel (UZ Brussel), Brussels, Belgium; 3 Hôpital Universitaire des Enfants Reine Fabiola (HUDERF), Brussels, Belgium; 4 Hôpital Universitaire Robert Debré, Paris, France; 5 Institut de Psychiatrie et de Neurosciences de Paris Inserm U894 Team 1, Paris, France; 6 Fondation SUSA-Université de Mons, Mons, Belgium; 7 PsyPluriel, Centre Européen de Psychologie Médicale, Brussels, Belgium; 8 Paris 8 Vincennes - St Denis University, Laboratoire de Psychopathologie et Neuropsychologie, Saint Denis, France; 9 Laboratory of Experimental Neurology, ULB, Brussels, Belgium; 10 National Fund of Scientific Research (FNRS), Brussels, Belgium; 11 Department of Neurology, Erasme Hospital, Brussels, Belgium; University at Buffalo, UNITED STATES

## Abstract

The overlap/distinctiveness between Attention-Deficit/Hyperactivity Disorder (ADHD) and Autism Spectrum Disorder (ASD) has been increasingly investigated in recent years, particularly since the DSM-5 allows the dual diagnosis of ASD and ADHD, but the underlying brain mechanisms remain unclear. Although both disorders are associated with brain volumetric abnormalities, it is necessary to unfold the shared and specific volume abnormalities that could contribute to explain the similarities and differences in the clinical and neurocognitive profiles between ADHD and ASD. In this voxel-based morphometry (VBM) study, regional grey matter volumes (GMV) were compared between 22 children with ADHD, 18 children with ASD and 17 typically developing (TD) children aged 8 to 12 years old, controlling for age and total intracranial volume. When compared to TD children or children with ASD, children with ADHD had a larger left precuneus, and a smaller right thalamus, suggesting that these brain abnormalities are specific to ADHD relative to ASD. Overall, this study contributes to the delineation of disorder-specific structural abnormalities in ADHD and ASD.

## Introduction

Attention-Deficit/Hyperactivity Disorder (ADHD) and Autism Spectrum Disorder (ASD) are two neurodevelopmental disorders with distinct core diagnostic criteria that often co-occur [1]. ADHD is characterized by impairing levels of inattention, and/or hyperactivity-impulsivity. ASD is defined by impaired social communication and interaction, and restricted, repetitive patterns of behaviour, interests or activities [1]. The DSM-5 (*Diagnostic and Statistical Manual of Mental Disorders*, Fifth Edition) allows for a comorbid diagnosis of ADHD and ASD, which was not permitted in previous editions, boosting research on the overlap and distinctiveness between these disorders [2]. Both disorders have been associated with executive function deficits [3], and neuroimaging studies have provided consistent evidence showing that clinical symptoms and cognitive deficits in these disorders are associated with underlying structural and functional brain abnormalities [4, 5]. Voxel-based morphometry (VBM) studies have evidenced volumetric abnormalities in both ADHD and ASD [5, 6].

In ADHD, reduced total brain and total grey matter volume (GMV) is a consistent finding according to meta-analyses of MRI volumetric studies [6–8]. Volumetric abnormalities have been found in prefrontal and other frontal areas, in the occipital and parietal lobes, and in the basal ganglia [7–10]. Basal ganglia volume reductions seem to be the most consistent finding across studies, more prominently in the lentiform nucleus and the caudate, in patients with ADHD compared to typically developing (TD) individuals [7, 9, 11, 12]. In a recent multisite analysis exploring subcortical brain volumes in 1713 participants with ADHD and 1529 TD individuals aged 4 to 63 years, Hoogman et al. [6] found significantly smaller volumes for the accumbens, the amygdala, the caudate, the hippocampus, and the putamen, bilaterally in the ADHD group, although effect sizes were small (from d = –0.10 to -0.19). Volume differences were most prominent in children, and non-significant in adults, suggesting a “normalization” of subcortical volume abnormalities with increasing age [6, 7]. In fact, brain volumetric reductions in ADHD have been interpreted as a maturational delay, as there is evidence for delayed peak volumes and cortical thickness in ADHD [6, 13].

ASD has been repeatedly associated with early modest but significant macrocephaly, followed by an abnormally slow or arrested brain growth and possibly a decrease in brain volume at older ages [14, 15]. In the largest VBM study to date in ASD (including 833 children and adults), Riddle et al. [16] found a subtle increase of approximately 1–2% in total brain and grey matter volumes in the ASD group compared to the TD group across all ages. Increased GMV in the left anterior superior temporal gyrus was the only regional abnormality detected in the ASD group. This result is consistent with previous meta-analyses showing volume abnormalities in the temporal lobe [5, 17–19]. Nevertheless, the study of regional volume abnormalities in ASD is mostly marked by inconsistencies across the literature, with studies showing increased GMV in some areas and volume reductions in other regions [5, 17–20].

While brain volume abnormalities in ADHD and ASD have been mostly investigated separately, only a limited number of studies have directly compared individuals with ADHD, ASD and/or comorbid ASD and ADHD (Table 1), in order to delineate the shared and specific structural abnormalities between these disorders [21–24]. ASD-specific volume abnormalities evidenced in these studies include: increased GMV in the right supramarginal gyrus [21], the left middle/superior temporal gyrus [22], and the left postcentral and right precentral gyri [23]. Decreased GMV in the left inferior frontal gyrus [24] and in the right posterior cerebellum [22] seems to be specific to ADHD.

**Table 1:** Volumetric studies comparing individuals with ASD and ADHD/ASD+ADHD.

Reference	n per group	Mean age in years (sd)	Mean IQ (sd)	Main results
Brieber [21]	15 ADHD	13.13 (1.4)	104.1(15.8)	ASD, ADHD>TD in left inferior parietal gyrus and postcentral gyrus/ ASD, ADHD<TD in left medial temporal lobe/ ASD>TD, ADHD in right supramarginal gyrus
	15 ASD	14.2(1.9)	106.8(21.4)	
	15 TD	13.3(1.8)	107.7(12.7)	
Lim [22]	44 ADHD	13.6(1.87)	92.2(11.7)	ADHD<ASD,TD in right posterior cerebellum/ ASD>TD, ADHD in left MTG/STG
	19 ASD	14.9(1.86)	113(15.7)	
	33 TD	14.3(2.52)	110(11.5)	
Mahajan [23]	30 ASD	10.5(1.7)	102(14)	ASD>TD,ASD+ADHD in right M1, left S1/ ASD+ADHD>TD in left IPC
	23 ASD+ADHD	10.3(1.4)	103(17)	
	63 TD	10.5(1.3)	112(11)	
Nickel [24]	30 ADHD	9.89(2.18)	93.71(13.32)	Significant main effect of the factor ADHD for the left Pars orbitalis volume (volume reduction)
	14 ASD	10.35(2.47)	93.71(14.17)	
	15 ASD+ADHD	10.32(2.21)	98.24(13.37)	
	36 TD	9.86(2.33)	97.79(13.05)	

ADHD: Attention-Deficit/Hyperactivity Disorder; ASD: Autism Spectrum Disorder; TD: typically developing; sd: standard deviation; MTG: middle temporal gyrus; STG: superior temporal gyrus; M1: primary motor cortex (precentral gyrus); S1: primary somatosensory cortex (postcentral gyrus); IPC: inferior parietal cortex.

Some studies have explored the association between ADHD or ASD symptom severity and regional grey matter volumes. O’Dwyer et al. [25] found that high ASD ratings were associated with a larger left caudate nucleus volume and a smaller left globus pallidus in participants with ADHD, unaffected siblings and TD participants. In a group of healthy adults, Geurts et al. [26] found that ASD symptom severity was correlated with left posterior cingulate volume, while ADHD symptom severity was associated with the volume of the right parietal lobe, the right temporal frontal cortex, the thalamus, and the left hippocampus/amygdala complex. Additionally, the volume of the left inferior frontal gyrus was correlated with symptom severity of both ASD and ADHD.

Overall, there is still an important heterogeneity and inconsistency across VBM findings, possibly explained by the heterogeneous character of the disorders themselves, but also by differences in sampling and data acquisition and analysis between studies [20]. The identification of shared and specific volume abnormalities between ASD and ADHD in carefully selected samples may contribute to understanding the clinical and neurocognitive similarities and differences between these disorders.

In this VBM study, total brain, grey matter and white matter volumes, and regional grey matter volumes were compared between children with ADHD, children with ASD and TD children, aged 8–12 years old. Based on previous literature, we hypothesized that: (1) children with ADHD would exhibit decreased total brain and grey matter volume, whereas the ASD group would exhibit slightly larger total brain and grey matter volumes; (2) the ASD group would show disorder-specific regional brain volume abnormalities in the temporal lobe and motor areas, and the ADHD group in the left inferior frontal gyrus and cerebellar regions.

## Materials and methods

### Participants

Seventy-one right-handed children aged 8–12 years were initially enrolled. Fourteen participants were excluded a posteriori: one after the fortuitous discovery of a brain anomaly during the MRI scan and 13 participants because of poor image quality. Exclusion criteria were: history of prematurity, neurological disorders, genetic disease, complications during labour with neonatal care unit hospitalization, disabling somatic pathology, left-handedness and contraindications to MRI. The final sample consisted of 22 children with ADHD, 18 children with ASD and 17 TD children. Children with ADHD were recruited at the Erasme Hospital Department of Neurology and PsyPluriel-Pastur in Belgium. Children with ASD were recruited in autism reference centres at the Queen Fabiola Children’s University Hospital and the SUSA Foundation in Belgium. TD children participated upon announcement or personal query. ADHD and ASD diagnoses were established by trained child psychiatrists according to the DSM-5 criteria [1]. All children with ADHD met the full Kiddie-Sads-Present and Lifetime Version K-SADS-PL [27] criteria for ADHD (combined-type). ASD diagnosis was established using the Autism Diagnostic Interview-Revised [28], the Autism Diagnostic Observation Schedule [29] and/or the Childhood Autism Rating Scale [30]. All participants had a General Ability Index higher than 70 on the Wechsler Intelligence Scale for Children–4th Edition [31, 32]. The parents of all participants filled the ADHD Rating Scale-IV (ADHD RS-IV [33]). Psychiatric comorbidities were assessed using the K-SADS-PL [27]. In the ADHD group, all children were medication-naïve and did not present any psychiatric comorbidity. In the ASD group, twelve participants had a comorbid ADHD diagnosis, but did not present any other comorbidity. Also in the ASD group, one participant had a history of methylphenidate and risperidone intake, one was currently taking psychostimulant medication but underwent a 24-hour washout period before the MRI scanning, and another participant was taking aripiprazole at the time of the scanning.

All participants and their parents gave signed informed consent to participate in this study approved by the Ethics Committee of the ULB-Erasme University Hospital, Belgium (P2014/120; CCB: B406201420437), and received 50 euros to cover transportation expenses.

### Anatomical image acquisition

Brain imaging data was collected using a Discovery MR750w 3.0T scanner (GE Medical Systems, Milwaukee, Wisconsin, USA) at UZ Brussel. Anatomical images were obtained using a T1-weigthed sagittal 3D TFE (turbo field echo) sequence: repetition time = 8.644 ms, echo time = 3.244 ms, inversion time = 450 ms, flip angle = 12°, field of view = 240x240 mm^2^, matrix size = 256 x 256 x 128 and voxel size = 0.94x0.94x1.2 mm3.

### Image quality control and pre-processing

Imaging data was analysed using the Computational Anatomy Toolbox (CAT12; http://www.neuro.uni-jena.de/cat12/CAT12-Manual.pdf), implemented in SPM12 (http://www.fil.ion.ucl.ac.uk/spm/), executed in MATLAB (R2019a, The MathWorks, Inc., Natick, MA). CAT12 workflow was adapted for paediatric samples, using customized tissue probability maps (TPM) created with the template-o-matic toolbox (TOM8; https://irc.cchmc.org/software/tom/downloads.php) and a customized DARTEL (Diffeomorphic Anatomical Registration Through Exponentiated Lie Algebra) template.

T1 images were visually inspected for motion, gross anatomical artefacts and adequate whole-brain coverage. Images were excluded if their weighted average Image Quality Rating (IQR) was lower than 80%, corresponding to a “good” image quality (CAT12; http://www.neuro.uni-jena.de/cat12/CAT12-Manual.pdf). Mean correlation between all volumes was visualized through CAT12. Volumes with a correlation below two standard deviations from the sample mean were again visually inspected for artefacts. Thirteen scans were excluded due to poor image quality. IQR did not significantly differ between the ASD, ADHD and TD children groups (F(2,54) = 0.35, p = 0.70).

Following quality control, customized TPMs were created using TOM8 with a matched pairs approach including vectors for age and sex [34]. Next, affine registered white matter and grey matter tissue segments were extracted to construct a customized DARTEL template, registered to the MNI (Montreal Neurological Institute) -ICBM (International Consortium for Brain Mapping) space. Individual images were corrected for bias-field inhomogeneities, segmented and modulated normalized using the customized TPMs and the DARTEL template created in previous steps. Finally, the normalized grey matter segments were smoothed using a Gaussian filter (5 mm full-width half-maximum). Total intracranial volume (TIV) was calculated for each participant using CAT12.

### VBM statistical analysis

Analysis of variance (ANOVA) was used to compare total brain volume, total GMW and total white matter volume (WMV) in cm^3^ between our three groups, using IBM SPSS Statistics version 23 (IBM Corp., Armonk, N.Y., USA). Total brain volume (TBV) was calculated as the sum of total GMV and total WMV.

Individual subject smoothed grey matter volumes were entered into a second level analysis to estimate between-group differences using a one-way ANOVA. Global scaling was used with TIV and age was entered as a covariate. All analyses were conducted without IQ as a covariate [35]. Threshold-Free Cluster Enhancement technique TFCE [36] was conducted using the TFCE Toolbox Version 183 1.0 (http://dbm.neuro.uni-jena.de/tfce/) with 5000 permutations. Results were considered significant at TFCE-FWE-corrected p = 0.05 and the p-value was Bonferroni-adjusted for the number of planned comparisons (ADHD>ASD, ASD>ADHD, ASD>TD, TD>ASD, ADHD>TD, TD>ADHD): p = 0.05/6 = 0.008. Anatomical labelling of brain regions was performed using the Anatomy Toolbox [37] and the Brainnetome Atlas [38].

Extraction of mean cluster intensity was performed using the marsbar toolbox (http://marsbar.sourceforge.net/) and plotted using RStudio (http://www.rstudio.com/). When between-group differences in a region were shown to be disorder-specific, correlations between the ADHD RS-IV total score and mean cluster intensities in that region extracted using the marsbar toolbox (http://marsbar.sourceforge.net/) were examined. VBM results visualization was performed using MRIcroGL (http://www.cabiatl.com/mricrogl/).

## Results

### Participant characteristics

Sex was not significantly related to diagnosis and groups did not differ on age (see Table 2). IQ was significantly higher in the TD group compared to either the ADHD (p = 0.003) or the ASD group (p = 0.012). Groups differed significantly on the ADHD RS-IV Total, Inattention and Hyperactivity scores (all ps<0.001). Additional demographic data analyses fractionating the ASD group in terms of the presence or absence of comorbid ADHD are reported in S1 Table.

**Table 2:** Demographic data of the participants.

	ADHD (n = 22)		ASD (n = 18)		TD (n = 17)		Between-group difference			Post hoc
	**M/F**		**M/F**		**M/F**		***χ2***	***df***	***p***	
**Sex**	16/6		16/2		12/5		2.07	2	0.36	ns
	***M***	***SD***	***M***	***SD***	***M***	***SD***	***H***	***df***	***p***	
**Age (months)**	122	18.51	125.11	16.29	126.12	19.48	0.59	2	0.74	ns
**IQ**	100.23	13.74	100.22	16.73	118.59	14.93	12.44	2	0.002	ADHD, ASD<TD
**ADHD RS-IV**										
Total	33.91	9.96	21.94	8.71	7.35	4.8	37.31	2	<0.001	ADHD>ASD>TD
Inattention	19.27	4.38	13.67	6.71	4.18	2.67	32.66	2	<0.001	ADHD, ASD>TD
Hyperactivity-Impulsivity	14.64	7.32	8.28	4.16	3.18	3.4	25.38	2	<0.001	ADHD, ASD>TD

ADHD: Attention-Deficit/Hyperactivity Disorder; ASD: Autism Spectrum Disorder; TD: typically developing; M/F: Male/Female; χ2: Pearson’s Chi-squared test; M: mean; SD: Standard deviation; H: test statistic for the Kruskal-Wallis test; df: degrees of freedom; p: p-value; ns: not significant; ADHD RS-IV: ADHD Rating Scale-IV.

### Analysis of total brain and total GMV/ WMV

Groups did not significantly differ on total brain volume and total GMV/WMV (see Table 3). Although total brain and total grey matter volumes were numerically lower for the ADHD group compared to the TD group, in line with previous literature, the difference failed to reach significance. Across all participants, there was a positive significant correlation between IQ and total GMV (r_s_ = 0.31, p = 0.020), total WMV (r_s_ = 0.35, p = 0.008) and TBV (r_s_ = 0.32, p = 0.015). Age was not significantly associated with total GMV, but there was a positive significant correlation between age and total WMV (r_s_ = 0.37, p = 0.005), and between age and TBV (r_s_ = 0.29, p = 0.031) across all participants. Age could not be entered as a covariate in this between-group comparison analysis because the data violated the ANCOVA assumption known as homogeneity of regression slopes: there was a significant interaction between diagnostic group and age on total GMV (p = 0.019), total WMV (p = 0.012) and total brain volume (p = 0.009), evidencing that the relationship between age and these volume measures differ between groups. Additional between-group analyses fractionating the ASD group in terms of the presence or absence of ADHD comorbidity are reported in S2 Table.

**Table 3:** Between-group differences in TBV and total GMV/WMV.

	ADHD (n = 22)		ASD (n = 18)		TD (n = 17)		Between-group difference			Post hoc
	*M*	*SD*	*M*	*SD*	*M*	*SD*	*F*	*df*	*p*	
**Total GMV (cm**^**3**^**)**	725.85	67.96	717.82	49.44	743.68	71.47	0.75	2,54	0.48	ns
**Total WMV (cm**^**3**^**)**	466.16	60.9	471.77	52.33	502.03	50.38	2.22	2,54	0.12	ns
**TBV (cm**^**3**^**)**	1192	120.79	1189.59	95.73	1245.72	116.3	1.43	2,54	0.25	ns

ADHD: Attention-Deficit/Hyperactivity Disorder; ASD: Autism Spectrum Disorder; TD: typically developing; M: mean; SD: Standard deviation; F: test statistic for ANOVA; df: degrees of freedom; p: p-value; ns: not significant; GMV: grey matter volume; WMV: white matter volume; TBV: total brain volume.

### VBM analysis of regional grey matter volume differences

**Fig 1:**
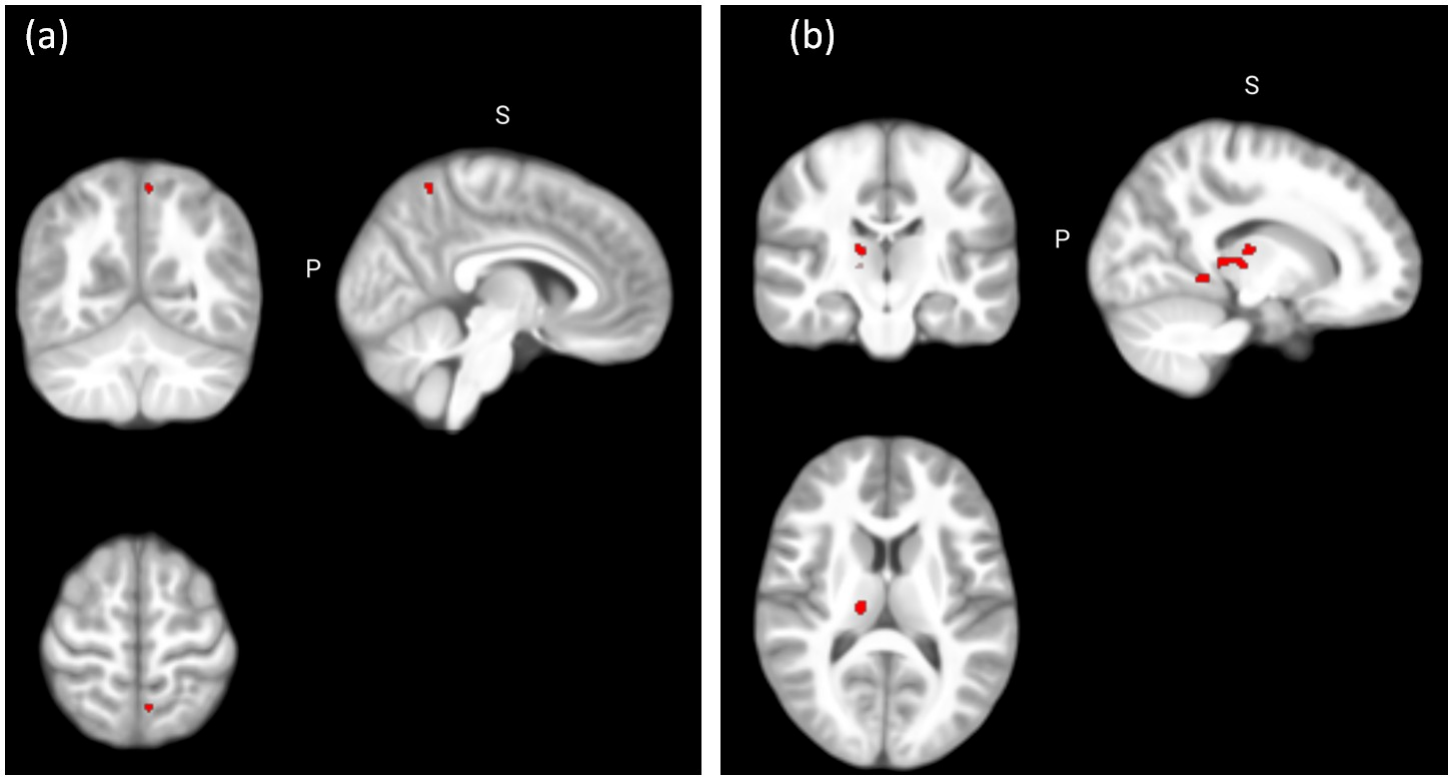
Between-group differences in regional grey matter volume. (a) Significant larger grey matter volume in the ADHD group compared to the TD group in the left precuneus (x = -6, y = -58, z = 63) at p < 0.05 (FWE TFCE corrected, whole brain analysis); (b) Significant decreased grey matter volume in the ADHD group compared to the TD group in the right thalamus (x = 15, y = -20, z = 12) at p < 0.05 (FWE TFCE corrected, whole brain analysis).

Whole-brain analyses disclosed larger GMV in the left precuneus and the right cerebellum (lobules IV-V) in children with ADHD compared to TD children; and in the left cerebellum (lobules IV-V) in children with ADHD compared to children with ASD (p<0.05; FWE-TFCE corrected). Children with ADHD displayed decreased GMV in the right parahippocampal gyrus and the right thalamus compared to the TD group (p<0.05, FWE-TFCE corrected; Table 4; Fig 1). Nevertheless, these did not survive Bonferroni correction for the number of planned comparisons. No significant differences were found between children with ASD and TD children at p<0.05 (FWE-TFCE corrected).

**Table 4:** Between-group differences in regional grey matter volumes.

Contrast	Hemisphere	Anatomical region	MNI coordinates			k	p
			x	y	Z		
**Whole brain**							
ADHD>ASD	L	Cerebellum (IV-V)	-20	-36	-24	19	0.046*
ADHD>TD	L	Precuneus	-6	-58	63	18	0.041*
	R	Cerebellum (IV-V)	30	-28	-30	12	0.046*
TD>ADHD	R	Parahippocampal gyrus	18	-39	-6	76	0.030*
	R	Thalamus	15	-20	12	148	0.036*

ADHD: Attention-Deficit/Hyperactivity Disorder; ASD: Autism Spectrum Disorder; TD: typically developing; k: cluster size; p: p-value.

*Significant at p < 0.05 (FWE TFCE corrected, whole-brain analysis).

### Disorder-specificity of grey matter volume abnormalities

**Fig 2:**
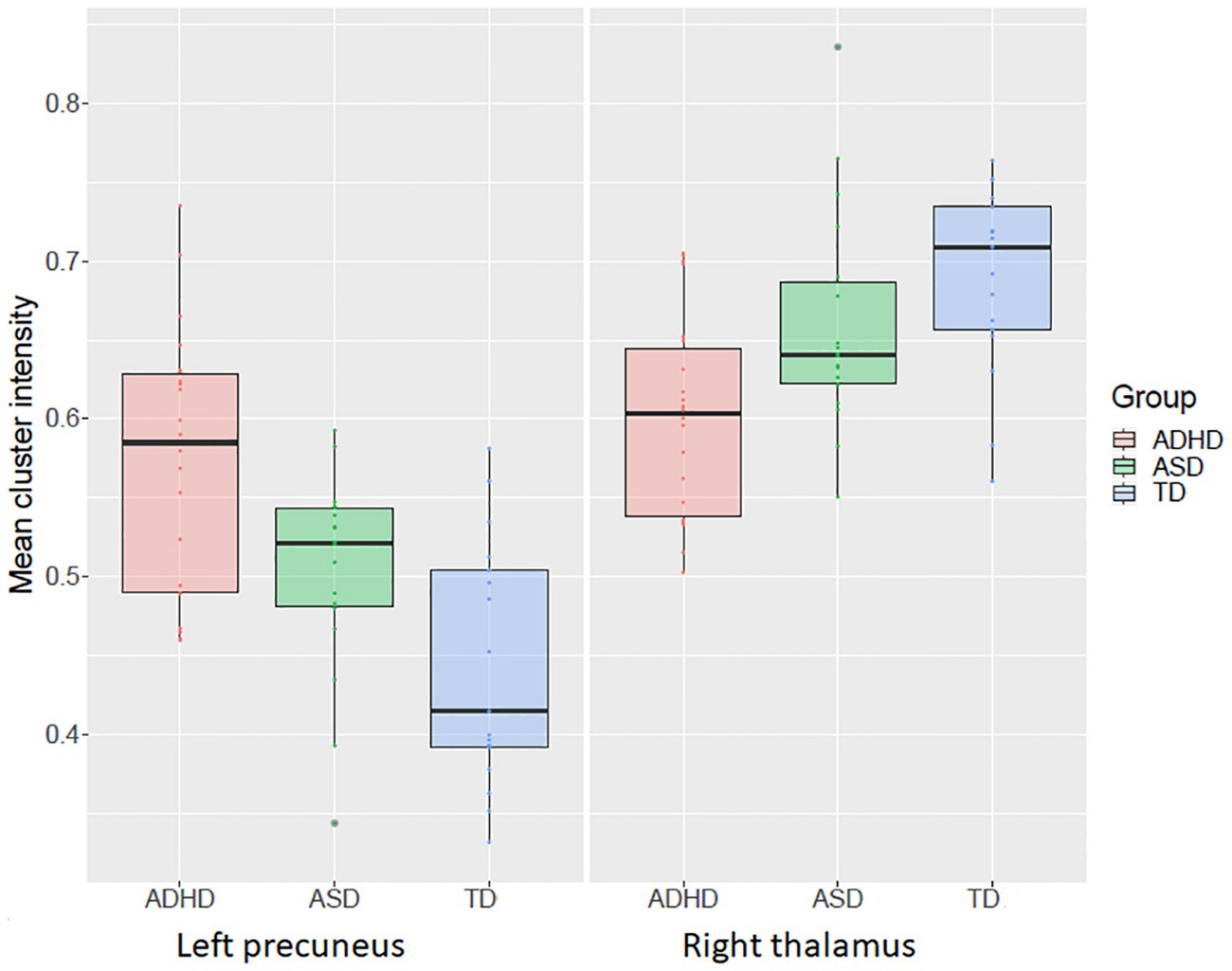
Mean cluster intensity in the left precuneus and the right thalamus. Significant between-group difference in GMV in the left precuneus and the right thalamus at p < 0.05 (FWE-TFCE corrected, whole brain analysis).

In order to explore the disorder-specificity of our results with regard to ASD or ADHD, regions of interest were created using 6 mm-radius spheres around the MNI coordinates in which significant between-group differences were disclosed, and the three groups were compared (see Table 4). The initial voxel threshold was set to 0.001 at the whole brain level uncorrected and multiple testing was accounted for on voxel level based on a p^FWE-SVC^ of 0.05. ROI analysis revealed larger GMV in the left precuneus in the ADHD group compared to the ASD group, suggesting increased GMV in the left precuneus is specific to ADHD relative to ASD (p^FWE-SVC^ = 0.018; x = -11, y = -60, z = 66, 7 voxels). ROI analysis also revealed a decrease in GMV in the right thalamus in the ADHD group compared to the ASD group (p^FWE-SVC^ = 0.025; x = 17, y = -20, z = 11, 9 voxels), suggesting decreased GMV in the right thalamus is specific to children with ADHD relative to ASD (Fig 2).

**Fig 3:**
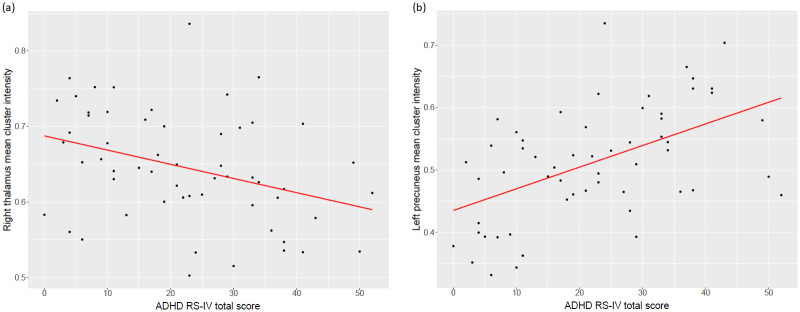
Correlation between ADHD RS-IV total score and mean cluster intensity. (a) Mean cluster intensity in the right thalamus, (b) Mean cluster intensity in the left precuneus. Values were extracted using the marsbar toolbox.

Finally, correlation analyses conducted across all participants evidenced a significant negative correlation between the ADHD RS-IV total score and mean cluster intensity in the right thalamus (r = -0.35, p = 0.008), and a significant positive correlation between the ADHD RS-IV total score and the mean cluster intensity in the left precuneus (r = 0.52, p<0.001). However, the associations did not hold within the groups (Fig 3).

## Discussion

This study contributes to the investigation of disorder-specific grey matter volume abnormalities in ASD and ADHD. Decreased GMV in the right thalamus and hippocampal gyrus, as well as increased GMV in the left precuneus and the right cerebellum were found in the ADHD group when compared to the TD group. Additionally, increased GMV in the left cerebellum was found in the ADHD group compared to the ASD group. Post-hoc analysis confirmed that decreased GMV in the right thalamus and increased GMV in the left precuneus were found in the ADHD group when compared to both the TD and the ASD groups, suggesting these volume abnormalities may be specific to ADHD.

The finding of an ADHD-specific decreased GMV in the right thalamus is consistent with a previous meta-analysis of VBM studies in ADHD [7], and extends this previous finding by showing this thalamic abnormality is ADHD disorder-specific relative to ASD. A recent VBM multisite analysis including approximately 1500 children (aged 4–14 years old) did not found significant differences for the thalamus volume between children with ADHD and TD children [6]. Nevertheless, fractional polynomial analyses in this cohort did show different developmental trajectories for the thalamus volume between patients with ADHD and TD participants. The between-group difference in the thalamus found in our more age-restrained sample suggests that abnormalities in this structure in ADHD may be restricted to specific time points in the developmental course. The thalamus is a key subcortical structure serving as an integrator and relay for multimodal signals to the cortex, and the synchronization of activity across multiple regions [39]. It is engaged by tasks requiring multiple cognitive functions, including attentional processes such as selective attention, regulating states of arousal and alertness, information processing and working memory [39, 40], functions often impaired in ADHD. At the functional level, abnormal BOLD activity in the right thalamus has been evidenced in samples with ADHD compared to TD participants during the execution of inhibition and attentional tasks [41, 42]. At the structural level, abnormalities in white matter microstructure in anterior thalamic radiations and posterior thalamic radiations have been reported in DTI studies [43–45]. Although the role of the thalamus in ADHD is sometimes overlooked, recent evidence and the present results puts the thalamus at the centre of the pathophysiology of ADHD [46].

The ADHD-specific increased grey matter volume in the left precuneus is also consistent with Nakao et al. [7], who showed a larger volume in this region in ADHD compared to TD individuals. Structural and functional studies have previously reported GMV abnormalities and abnormal functional brain activity in the precuneus. Still, results in this region are inconsistent in terms of the direction of the effect and the hemisphere involved. The first VBM study comparing a group of adolescents with ADHD versus ASD [21] evidenced larger GMV in the left precuneus in the ADHD compared to the TD group, but not when compared to the ASD group. In their study, contrary to ours, the ADHD and the ASD groups did not differ with respect to the parental rating of ADHD symptoms, possibly explaining the absence of differences between these groups in this region. In a recent study including children with ASD, children with ADHD and children with comorbid ASD and ADHD, Nickel et al. [24] showed a main effect of ADHD diagnosis on trend level in the right precuneus in terms of volume reduction, contrary to our result. Findings therefore remain largely inconsistent. In terms of brain activity in this region, decreased activation in the right precuneus in patients with ADHD relative to TD participants has been evidenced during the execution of attentional tasks in a meta-analysis of fMRI studies [41]. The authors explained this finding by pointing out the role of the precuneus in the representation of and orienting toward spatial locations. On the other hand, increased activation in the bilateral precuneus was found in ADHD relative to TD children during the execution of timing tasks in another meta-analysis of fMRI studies [47]. The authors proposed that this increased activation likely reflected problems deactivating the default mode network. Overall, this inconsistency in findings may be due to the complexity of this structure. The precuneus is part of the parietal cortex and is involved in a wide range of cognitive processes. Different functional subregions have been identified in this structure [48]. According to the Brainnetome Atlas [38], our results were more specifically located in a medial area of the precuneus also called “Pep” [49]. According to Fan et al. [38], this region in particular has been observed to be significantly activated by paradigms associated with visual distraction and attention, functions that are impaired in ADHD [50].

Other volume abnormalities were evidenced in the ADHD and the ASD groups. Nevertheless, we could not prove these were disorder-specific. Our correlational analyses support the disorder-specificity of our findings, since ADHD severity was significantly associated with mean cluster intensity in the right thalamus and the right precuneus. However, the associations did not hold within the groups. These results suggest that the reduction of the right thalamus and the enlargement of the left precuneus are associated with the categorical ADHD diagnosis rather than with symptom severity.

The findings in this study should be considered in the light of some limitations. The final sample was relatively small due to the exclusion of poor-quality scans, decreasing therefore the likelihood of biased results. This exclusion comes at a cost as head motion can be associated with clinical traits and the scans of the most severely impaired participants might therefore have been excluded, possibly inducing a sampling bias. Our sample was however large enough to detect robust significant between-group differences in grey matter volumes. One of the strengths of our study is that all children with ADHD were non-comorbid and medication naïve and therefore in this study, volume abnormalities found in the ADHD group cannot be confounded with medication or comorbidity effects. However, in the ASD group, three participants had a history of medication intake or were currently under medication, potentially confounding our findings. Finally, there was a high percentage of children in the ASD group who presented an ADHD comorbidity, consistent with recent comorbidity reports [51, 52], but which could potentially have a confounding effect in our findings. However, the severity of ADHD symptoms measured by the ADHD RS-IV was significantly higher in the ADHD group compared to the ASD group. Future work should continue exploring the commonalities and differences in regional GMV abnormalities between ASD and ADHD in a larger sample of children with ASD without comorbid ADHD and a larger sample of children with ASD with comorbid ADHD, as compared to children with the ADHD diagnosis only. Future studies are also encouraged to investigate whether these volume abnormalities are absolute differences between groups or reflect a maturational anomaly, since previous literature has highlighted the importance of the developmental aspect in volume abnormalities in these disorders.

## Conclusions

This study contributes to the delineation of disorder-specific structural abnormalities in ADHD and ASD. Our results evidenced that children with ADHD had a disorder-specific larger grey matter volume in the left precuneus and decreased grey matter volume in the right thalamus.

## Supporting information

S1 Table Demographic data of participants included in the analysis, fractionating the ASD group in terms of the presence or absence of ADHD comorbidity. ADHD: Attention-Deficit/Hyperactivity Disorder; ASD: Autism Spectrum Disorder; ASD only: ASD without comorbid ADHD; ASD+ADHD: ASD with comorbid ADHD; TD: typically developing; M/F: Male/Female; χ2: Pearson’s Chi-squared test; df: degrees of freedom; p: p-value; M: mean; SD: Standard deviation; H: test statistic for the Kruskal-Wallis test; ns: not significant; ADHD RS-IV: ADHD Rating Scale-IV.
(PDF)

S2 Table Between-group differences in total brain and total GM/WM volumes, fractionating the ASD group in terms of the presence or absence of ADHD comorbidity. ADHD: Attention-Deficit/Hyperactivity Disorder; ASD: Autism Spectrum Disorder; ASD only: ASD without comorbid ADHD; ASD+ADHD: ASD with comorbid ADHD; TD: typically developing; M: mean; SD: Standard deviation; F: test statistic for ANOVA; df: degrees of freedom; p: p-value; ns: not significant; GMV: grey matter volume; WMV: white matter volume; TBV: total brain volume.
(PDF)
