# Supplementary material for: Disorder-specific brain volumetric abnormalities in Attention-Deficit/Hyperactivity Disorder relative to Autism Spectrum Disorder
Source: PLoS One. 2020 Nov 9;15(11):e0241856. doi: 10.1371/journal.pone.0241856 (PMC7652272; doi:10.1371/journal.pone.0241856)
Supplement: S1 Table — ADHD: Attention-Deficit/Hyperactivity Disorder; ASD: Autism Spectrum Disorder; ASD only: ASD without comorbid ADHD; ASD+ADHD: ASD with comorbid ADHD; TD: typically developing; M/F: Male/Female; χ2: Pearson’s Chi-squared test; df: degrees of freedom; p: p-value; M: mean; SD: Standard deviation; H: test statistic for the Kruskal-Wallis test; ns: not significant; ADHD RS-IV: ADHD Rating Scale-IV. (PDF) [file pone.0241856.s001.pdf]

S1 Table. Demographic data of participants included in the analysis, fractionating the ASD group in terms of the presence or absence of ADHD comorbidity.

	ADHD (n=22)		ASD only (n=6)		ASD+ADHD (n=12)		TD (n=17)		Between-group difference			Post hoc
	**M/F**		**M/F**		**M/F**		**M/F**		***χ2***	***df***	***p***	
**Gender**	12/6		5/1		11/1		12/5		2.23	3	0.53	ns
	M	***SD***	***M***	***SD***	***M***	***SD***	***M***	***SD***	***H***	***df***	***p***	
**Age**	122.00	18.51	126.33	17.57	124.50	16.40	126.12	19.48	0.62	3	0.89	ns
**IQ**	100.23	13.74	98.17	18.86	101.25	16.35	118.59	14.93	12.44	3	0.006	ADHD<TD
**ADHD RS-IV**												
Total	33.91	9.96	13.00	5.33	26.42	6.24	7.35	4.80	41.45	3	<0.001	ADHD>ASD only, TD/ ASD+ADHD>TD
Inattention	19.27	4.38	6.50	4.28	17.25	4.39	4.18	2.67	39.79	3	<0.001	ADHD> ASD only,TD/ ASD+ADHD>ASD only,TD
Hyperactivity	14.64	7.32	6.50	3.08	9.17	4.45	3.18	3.40	26.21	3	<0.001	ADHD, ASD+ADHD>TD

ADHD: Attention-Deficit/Hyperactivity Disorder; ASD: Autism Spectrum Disorder; ASD only: ASD without comorbid ADHD; ASD+ADHD: ASD with comorbid ADHD; TD: typically developing; M/F: Male/Female; χ2: Pearson's Chi-squared test; df: degrees of freedom; p: p-value; M: mean; SD: Standard deviation; H: test statistic for the Kruskal-Wallis test; ns: not significant; ADHD RS-IV: ADHD Rating Scale-IV.
