# Supplementary material for: Disorder-specific brain volumetric abnormalities in Attention-Deficit/Hyperactivity Disorder relative to Autism Spectrum Disorder
Source: PLoS One. 2020 Nov 9;15(11):e0241856. doi: 10.1371/journal.pone.0241856 (PMC7652272; doi:10.1371/journal.pone.0241856)
Supplement: S2 Table — ADHD: Attention-Deficit/Hyperactivity Disorder; ASD: Autism Spectrum Disorder; ASD only: ASD without comorbid ADHD; ASD+ADHD: ASD with comorbid ADHD; TD: typically developing; M: mean; SD: Standard deviation; F: test statistic for ANOVA; df: degrees of freedom; p: p-value; ns: not significant; GMV: grey matter volume; WMV: white matter volume; TBV: total brain volume. (PDF) [file pone.0241856.s002.pdf]

S2 Table. Between-group differences in total brain and total GM/WM volumes, fractionating the ASD group in terms of the presence or absence of ADHD comorbidity.

	**ADHD (n=22)**		**ASD only (n=6)**		**ASD+ADHD (n=12)**		**TD (n=17)**		**Between-group difference**			**Post hoc**
	M	*SD*	*M*	*SD*	*M*	*SD*	*M*	*SD*	*F*	*df*	*p*	
**Total GMV (cm^3)**	725.85	67.96	720.2	38.54	716.63	55.66	743.68	71.47	0.50	3,53	0.69	ns
**Total WMV (cm^3)**	466.16	60.9	483.76	36.02	465.77	59.37	502.03	50.38	1.61	3,53	0.20	ns
**TBV (cm^3)**	1192	120.79	1203.95	65.22	1182.4	109.82	1245.72	116.3	0.99	3,53	0.41	ns

ADHD: Attention-Deficit/Hyperactivity Disorder; ASD: Autism Spectrum Disorder; ASD only: ASD without comorbid ADHD; ASD+ADHD: ASD with comorbid ADHD; TD: typically developing; M: mean; SD: Standard deviation; F: test statistic for ANOVA; df: degrees of freedom; p: p-value; ns: not significant; GMV: grey matter volume; WMV: white matter volume; TBV: total brain volume.
